# Principle-based structured case discussions: do they foster moral competence in medical students? - A pilot study

**DOI:** 10.1186/s12910-017-0181-1

**Published:** 2017-03-03

**Authors:** Orsolya Friedrich, Kay Hemmerling, Katja Kuehlmeyer, Stefanie Nörtemann, Martin Fischer, Georg Marckmann

**Affiliations:** 1 Institute for Ethics, History and Theory of Medicine at Ludwig Maximilian University (LMU), Lessingstr. 2, 80336 Munich, Germany; 2 Institute for Pedagogic and Rehabilitation Psychology at the University of Leipzig, Neumarkt 9-19, 04109 Leipzig, Germany; 3 Institute for Medical Education at the University Hospital of LMU Munich, Lessingstr. 2, 80336 Munich, Germany

**Keywords:** Educational intervention, Medical ethics class, Medical students, Moral competence test, Moral competence

## Abstract

**Background:**

Recent findings suggest that medical students’ moral competence decreases throughout medical school. This pilot study gives preliminary insights into the effects of two educational interventions in ethics classes on moral competence among medical students in Munich, Germany.

**Methods:**

Between 2012 and 2013, medical students were tested using Lind’s Moral Competence Test (MCT) prior to and after completing different ethics classes. The experimental group (EG, *N* = 76) participated in principle-based structured case discussions (PBSCDs) and was compared with a control group with theory-based case discussions (TBCDs) (CG, *N* = 55). The pre/post C-scores were compared using a Wilcoxon Test, ANOVA and effect-size calculation.

**Results:**

The C-score improved by around 3.2 C-points in the EG, and by 0.2 C-points in the CG. The mean C-score difference was not statistically significant for the EG (*P* = 0.14) or between the two groups (*P* = 0.34). There was no statistical significance for the teachers’ influence (*P* = 0.54) on C-score. In both groups, students with below-average (M = 29.1) C-scores improved and students with above-average C-scores regressed. The increase of the C-Index was greater in the EG than in the CG. The absolute effect-size of the EG compared with the CG was 3.0 C-points, indicating a relevant effect.

**Conclusion:**

Teaching ethics with PBSCDs did not provide a statistically significant influence on students’ moral competence, compared with TBCDs. Yet, the effect size suggests that PBSCDs may improve moral competence among medical students more effectively. Further research with larger and completely randomized samples is needed to gain definite explanations for the results.

## Background

The practice of medicine is a fundamentally moral endeavor. Investigating moral judgment competence therefore is highly important in medicine. Hence, the moral development of medical students should be considered a high priority for medical educators. To evaluate medical students’ abilities of moral reasoning and moral competence, different methods with varying theoretical backgrounds regarding moral competence have been used, mostly utilizing or being derived from Kohlberg’s Moral Judgement Interview (MJI), but also Rest’s Defining Issues Test (DIT), or Gibbs’ Sociomoral Reflection Measure have been employed [1]. The theoretical perspectives and the subsequent discussions of the different instruments are too complex to be addressed and evaluated here. The Moral Competence Test (MCT), which we apply in this pilot study, is one of the instruments that have been used to measure moral competence in medical students [2, 3]. MCT aims to measure the ability to rate arguments based not on opinion-agreement, but on the moral quality of the arguments [3]. MCT uses the dual-aspect theory of Lind as a theoretical reference for moral development, where moral competence and orientation are aspects of moral judgment and where affects and cognition are two aspects of moral behavior [2].

Education has been described in the literature as the main factor that fosters moral competence [2]. Since moral competence is becoming increasingly important for medicine, it is particularly alarming that several studies with different instruments (with MCT [2–8], with MJI [9–11], and with DIT [12]) suggest that this competence, as well as moral reasoning ability, seem to decrease during medical school or do not increase to a similar extent as during other ongoing formal educational processes [1]. The findings that moral competence can decline are important to adjusting medical education in a way that allows for an increase of moral competence and for the ability of future medical professionals to better face morally challenging situations.

Results from different studies with MCT indicate that the regression of students’ moral competence could be explained by rare opportunities of taking responsibility and guided reflection, indicating an unfavorable learning environment during medical education [2, 6, 13]. Ethics courses during medical education could offer a better learning environment and some studies have already suggested that moral reasoning skills could be increased, especially if small-group discussions of moral dilemmas were involved [1, 14, 15]. Education in medical ethics should in addition be less theoretical but rather learner- and problem-based to foster moral reasoning skills [1]. Other studies indicate, however, that ethics teaching and case discussions in general do not significantly foster moral competence and moral reasoning skills [1, 2, 4, 16, 17]. Shorr et al. explain the lack of increase by the fact that medical students have already gained a certain ethical perspective by the time of their arrival at medical school [1, 17]. Results also show that although the moral attitudes of medical students indeed have reached a high level (they prefer the highest Kohlberg stages 5 and 6) when they enter university, their scores for competence in moral reasoning could be higher [3].

There are no concluding results for the question of whether and how ethics courses can increase moral competence in medical students. There is still a research gap for addressing different educational interventions in medical students, which consider previous positive results of fostering moral competence, but add further interventional variables and test the results with established instruments. We therefore aimed to test two different educational interventions, but coevally taking into account positive results of previous studies: Both educational interventions were conducted with small subgroups, with case discussions, with guided reflection and were learner- and problem-based.

In this pilot study, we compared the effects of two different educational interventions: 1) Principle-based structured case discussion (PBSCD) adopts well established principles of biomedical ethics in a systematic manner, it captures and balances different normative perspectives for the respective case and involves the participant strongly in the process of weighing up; 2) Theory-based case discussion (TBCD) provides the participants with a normative perspective that should be applied in a formalized way for solving the particular case.

We consider ethical decision making as better justified in cases where the decisions are not only representations of opinions, but the results of weighing up the relevant moral and case specific aspects [18, 19]. The intervention in our study with PBSCD is based on this presupposition of justified ethical decision making and MCT is an instrument that can measure the grade of opinion free assessment of moral arguments. We *hypothesized* that moral competence in medical students could be enhanced more through PBSCDs than through TBCDs.

## Methods

### Participants

We included 131 medical students. The two groups had the same general features, being of similar age and students of medicine. The experimental group (EG), *N* = 76, consisted of four subgroups that received medical ethics classes with PBSCDs (14 males, 61 females, 1 missing data; age M = 22.3, SD = 3.8). The control group (CG), *N* = 55, consisted of three subgroups (23 males, 32 females; age M = 21.7, SD = 3.8) that participated in classes in medical ethics with TBCD. All subgroups of the EG and CG were in their first part of their medical education (pre-clinical curriculum, semesters 1–4), except EG_1_ (*N* = 9)_,_ which was offered as an elective course in the clinical part of the curriculum (semesters 8/9).

### Materials and design

#### Teaching method

The CG received lectures in philosophical theories that are considered relevant for medical ethics. Students learned the basics of metaethics, deontological and virtue ethics, consequentialism, and principlism. Each of the educated normative theories was applied to different dilemma cases as TBCD, gained from literature.

The chosen teaching method in the EG was PBSCD, which was developed by Marckmann [18, 19], based on McCullough and Ashton’s clinical handbook for medical ethics [20] and on the principles of biomedical ethics: autonomy, beneficence, non-maleficence, and justice (principlism) by Beauchamp and Childress [21]. As a first step, all medical options have to be identified and analyzed, which means identifying information on the patient (history, symptoms, social situation, diagnosis and prognosis) and developing management strategies (decisional options and their aims), as well as the outcome of each strategy (benefits and risks). As a second step, all moral obligations towards the patient are determined, evaluated, and specified if needed. These obligations are expressed in the principles of autonomy (patient perspective), beneficence, and non-maleficence (best interest perspective). Students have to decide on the preferable option considering that particular perspective. In the next step, the duties towards third parties, such as family or society, have to be discussed for each case with respect to the principle of justice, and the best option for this perspective has to be specified. In a fourth step, balancing these moral obligations is required. In the case of conflicting obligations, the justification to prioritize one obligation over the other is built on good, case-based reasons. A decision is to be made, and afterwards, the objections are reflected on to recognize potential mistakes, discover other decision options (sometimes based on other theories), and to determine how this type of conflict could have been avoided to prevent similar problems in the future [18, 20].

The theoretical background of PBSCD presumes justified ethical decision making as being not only representations of opinions, but the results of weighing up the relevant moral and case specific aspects of a case [18, 19]. The balancing of different relevant moral arguments, also independently from subjective intuition, is exercised in PBSCDs. Students in the EG are strongly confronted with a hypothetical patient and the responsibility to find an ethically justified result, which resembles neither their previous opinion nor a formalized application of an ethical theory.

#### Measurement and scoring

To measure moral competence we used the MCT developed by Georg Lind [22, 23]. The German version of the MCT was validated in respect to several analytical and empirical criteria (predictions derived by Kohlbergian research) and has been tested in different populations over the past 30 years [24–27]. Moral competence is defined in this test as the degree to which a person judges other people’s arguments consistently based on their own moral points of view, rather than based on agreement with their opinions on a given issue. According to this definition, integrated moral judgment behavior takes place if one’s guiding orientation applies not only to arguments defending one’s own opinion, but also to arguments opposing one’s own opinion. The focus of the MCT is to assess how a person deals with moral arguments, especially with counter-arguments [22, 23].

The MCT includes two moral dilemmas, a worker’s dilemma and a doctor’s dilemma. First, the participant is required to give his or her opinion regarding the behavior of the doctor and the worker on a Likert-type scale from -3 to +3. Then the participant has to rate six arguments in favor of and six arguments against their opinion on a Likert-type scale from “I strongly reject” (-4) to “I strongly accept” (+4). The six arguments are based on the moral stages defined by Kohlberg [24–26].

We used the *C-score* as the main outcome parameter of our research. It measures the degree to which the pro and con arguments in the MCT are determined by moral points of view rather than opinion-agreement [27]. The C-score reflects the subject’s competence to weigh pro and contra moral arguments consistently and independently of his/her previously stated opinion [27]. It’s calculation is described in the literature [22].

The C-score can range from 0 to 100. A C-score of 1–9 is considered very low, 10–19 as low, 20–29 as medium, 30–39 as high, 40–49 as very high, and above 50 is considered extraordinarily high [22, 27]. C-scores are usually between 0 and 40 and cannot be feigned upward; in general, no gender differences in moral competence could be observed for the MCT [2, 22, 28].

#### Statistical analysis

We performed a paired Wilcoxon Test to compare the pre and post C-scores in the EG and an unpaired Wilcoxon Test for the comparison between the EG and the CG. We conducted an unpaired Wilcoxon Test within the CG to check if there is a correlation between the augmentation in C-scores and the different principal lecturers. We also conducted ANOVA with repeated measures to analyze the effects of the condition CG versus EG, time point pre versus post and of an interaction of both. The results were considered statistically significant at *P* < 0.05; a tendency was reported at *P* < 0.1. We used SPSS, Version 22 (IBM Ehningen, Baden-Wuerttemberg, Germany).

We also looked for Cohen’s d, an effect size calculated as the difference between the means, divided by the pooled standard deviation.

We also used the absolute effect-size (aES) to compare between groups for the purposes of this investigation. The aES is the difference between the means of two measures.

The advantages of absolute effect-sizes are that they are 1) independent of the number of measured values and 2) independent of the variability of the measured values [4]. The relevance of the aES depends on the context. On a 100-point broad scale, aES > 10 C-points (10% of the scale) can be considered relevant. Since the range of C-score results for the MCT is usually between 0 and 40 points, an aES of 4 C-points would be 10% of the relevant scale and, therefore, a relevant (but not necessarily statistically significant) result [13, 29].

### Procedure

The study was performed between January 2012 und August 2013 at the Institute for Ethics, History, and Theory of Medicine at Ludwig Maximilian University (LMU) Munich, Germany. Students participated in two different courses on medical ethics, each consisting of a total of 20 teaching units (45 min per unit). The courses were offered as an elective course within the medical curriculum at LMU Munich. Students who took the class took part in the study voluntarily (providing informed consent). The course assignment was dependent on the student’s preferences and availability. Recruitment to the CG and the EG was not completely randomized, but it happened quasi-randomly as the CG and the EG recruitment procedures took place (for groups in the same term) on the same day. There were no major content differences in the announcement of the ethics classes for any of the groups.

The first author (OF) was the principal lecturer for the EG_1_-EG_4_ and CG_1_ classes and the visiting lecturer for CG_2_ and CG_3,_ where a colleague from the same institute was the principal lecturer. OF is a lecturer in medical ethics and was introduced to the method of PBSCDs by its founder (GM). At the beginning of the study, she had 5 years of experience lecturing in ethics.

In the CG students held short presentations on different normative theories and the lecturer discussed the pros and cons of each theory and its applicability to medical ethics and to dilemma cases (>5) in TBCDs with them. TBCDs took about 60–90 min each.

The participants in the EG had guided discussions on five different moral dilemma cases in medicine with PBSCDs. The realistic cases were written by GM according to his experiences in clinical ethics consultations. Case discussions took 60–90 min each. The other teaching units in the EGs consisted of instruction on ethical theories comparable to the introductory course in the CG.

We conducted the same MCT in the beginning and at the end of the ethics courses (after 20 teaching units) in both the CGs and EGs. When the MCT is used repeatedly, special attention should be given to the phenomenon of re-testing fatigue [22]. Thus, we gave careful instructions to the participants to reduce test weariness.

## Results

The response rate was 89.7% = ([131/146] * 100) according to the number of valid questionnaires. No C-score was calculated for MCT questionnaires in which more than two arguments were omitted [22]. The mean C-score for all participants was M = 29.1 (Standard Deviation, SD = 17.2).

For the EG, the mean C-score improved considerably over the course (aES = 3.2), but was not statistically significant in the paired Wilcoxon Test (*P =* 0.14), which shows that PBSCDs do not foster moral competence significantly. In the CG, the C-score only improved slightly over the course (aES = 0.2). The influence of the principal lecturer on pre/post C-scores in the CG was not significant in the unpaired Wilcoxon Test (*P* = 0.54), which means that the lecturer might have had an influence on the different conditions in the CG and EG, but not in a statistically significant way. The difference in pre/post C-scores between the two groups was not statistically significant in the unpaired Wilcoxon Test (*P* = 0.34). This indicates that the hypothesis of this study, namely that PBSCDs foster moral competence more than TBCDs, cannot be proved with certainty. Similar can be said for the repeated measures ANOVA; this did not provide significant results for the effects of the condition CG versus EG (*P* = 0.14), the time points pre versus post (*P* = 0.17) and of an interaction of both (*P* = 0.24).

There were no statistically significant effects of segmentation between the two dilemma cases (*P* = 0.81) for pre and post comparison for the CG versus the EG, the pre-post segmentation difference in the CG were 5.3 C-points in the EG 3.8 C-points.

**Fig. 1:**
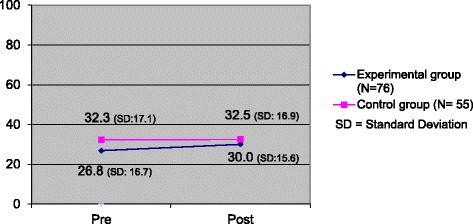
Pre/post difference in mean C-score, comparison between EG and CG

The relative effect-size was indicated by Cohen’s d = 0.21. The absolute effect-size (aES) of the C-score differences in the EG compared with the CG was 3.0 C-points (Fig. 1 and Table 1).

**Table 1:** Pre/post mean C-Scores and group sizes according to gender and prior ethics lessons

Group	N and C-Score	Time	Gender^a^		Prior ethics lessons	
			Male	Female	Yes	No
EG	N (76)		14	61	40	36
	C-Score	Pre	27.6	26.5	28.1	25.4
		Post	28.5	30.3	33.2	26.4
CG	N (55)		23	32	46	9
	C-Score	Pre	30.7	33.5	31.9	34.3
		Post	28.7	35.2	32.1	34.5

^a^One missing data

The pre C-score and the pre/post difference were not influenced by gender and prior experience with ethics lessons.

**Fig. 2:**
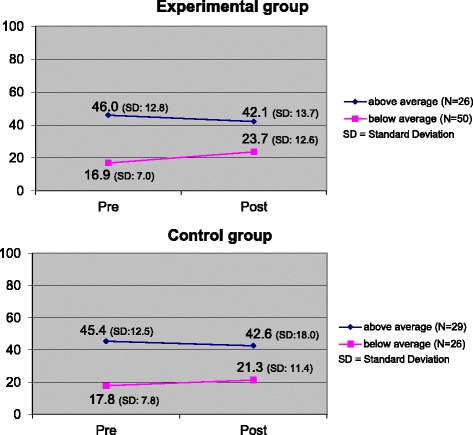
Pre and post C-Score grouped by condition (CG and EG) and students in comparison to their C-Score at the beginning

We divided the groups into participants with C-scores below and above average, according to the distribution of the overall average (M = 29.1, SD = 17.2), to examine how above and below average students performed. This division was necessary, because there was a significant pre C-score difference in the CG and EG in the Wilcoxon Test (*P* = 0.04), the pre C-score was higher in the CG than in the EG (+5.5 C-points). Without such a division less improvement in the CG could be attributed to a *ceiling effect*, where medical students who start with a high C-score cannot perform better (Fig. 2).

The unpaired Wilcoxon Test to compare the below-average groups in the CG and the EG pre-post did not provide statistically significant results (*P* = 0.38). For the above-average groups, there were no significant results as well (*P* = 0.54).

In the EG, the below-average group gained more points on the C-score (aES = 6.8) than did the below-average group in the CG (aES = 3.5). The absolute effect-size of the experimental condition compared with the control condition in the below-average group is 3.3, indicating a small effect. In both groups, participants regressed in moral competence if they started with above-average C-scores (aES_E_ = -3.9, aES_C_ = -2.8).

## Discussion

Several empirical studies suggest that the abilities of medical students in moral reasoning decline during their education [1]. Though there is a lack in consensus about the goals of education in medical ethics, it was suggested that the improvement of students’ skills in analyzing and resolving moral dilemmas is a key aspect [1]. There are conflicting results about the question of whether and which type of ethics courses are actually able to enhance these skills [1, 2, 4, 16, 30]. Though there are some aspects and tools of educational interventions in ethics courses that were identified as effective to improve moral reasoning abilities in medical students: small groups and case discussions for at least 20 h [1, 15, 30]; guided reflection [6, 13]; learner- and problem-based ethics education [1]. We aimed to find out in a pilot study, if PBSCDs provide an intervention that could foster moral competence in medical students in the EG, contrary to TBCDs in the CG. For both CG and EG, we have chosen small groups of ethics classes with case discussions, with 20 h education and both groups went through a process of guided reflection. The intervention difference between the CG and the EG was following: in the EG students had to consider, to specify and to balance different moral aspects and perspectives during PBSCDs with a high level of personal involvement to come to a conclusion; contrary to the CG, where they had to apply each with a moral theory to a given case in TBCDs in a formalized way. We did not choose a control group without any intervention (no ethics class) versus the EG, because such a control group would only have allowed for the interpretation - in case of C-score increase in the EG - that any ethics course or any case discussion is able to improve the C-score. Because we wanted to show the specific benefits of PBSCDs for C-score results, we compared PBSCDs in the EG with another intervention in ethics classes in the CG, which also includes a case discussion, but in a different manner, namely as TBCD.

The comparison of PBSCDs and TBCDs didn’t provide a statistically significant difference between the two ethics classes, but the effect size suggested a relevant difference between PBSCDs and TBCDs: Medical ethics classes with PBSCD were able to enhance moral competence of medical students in this study in contrast to those with TBCD. The deficient statistical significance could have been due to the relatively small number of participants as the effect size was small. Our results therefore allow only for insights into first attempts to apply PBSCD to foster moral competence.

The lack of proper randomization also impaired our statement. Though there is a pre-post comparison possible for the different interventions, as the choice of the CG and the EG happened quasi-randomly and the two groups had the same general features (age; all were students of medicine; students had the same days and recruitment procedures in the CG and EG in each term; there were no major content differences in the announcement of the ethics classes). The difference in the effect size could still be due to chance or a selection bias and further research with larger samples (due to the small effects), and complete randomization is needed to confirm this preliminary result and to examine which features explain the effects of PBSCDs on moral competence.

Furthermore, we could not avoid the initial difference between the C-scores of the two groups. A third variable could have influenced the students’ selection of one of the courses and could explain the differences between the starting conditions. In that gender and previous ethics lessons experience did not explain the high initial C-score in the CG, we divided both groups into two C-score subgroups to assess the potential influence of the different pre C-scores in the CG and the EG. Otherwise, a ceiling effect in the CG could explain our results. We were able to show that students with low and medium C-scores made a positive development both in the EG and the CG. Though we have no statistically significant results, we were able to show that below-average students in the EG achieved higher C-scores after the course than below-average students in the CG, since the absolute effect-size in below-average students was 3.3 C-points.

Further research is necessary to describe why C-score increases are specifically applicable to below-average C-score students. The absolute decline in C-scores from pre- to post-measurement in the above-average groups was not very large, but the reverse effect raises questions. So far, we have no definite explanation as to why students with above-average C-scores in both groups did not enhance their moral competence through these courses. One possibility is that it is more difficult to re-attain high or very high C-scores or even to enhance them through education. It is possible that repeated PBSCDs could become a tedious method for participants that already have high moral competence. An alternative explanation could be that test weariness is higher in participants with initially high moral competence. A scope for future teaching experiments could be to improve, or at least to stabilize, the moral competence of medical students with above-average C-scores.

Another limiting factor could be seen in the different educational stage for EG_1_ (*N* = 9) compared with the other subgroups, in that the participants in this subgroup were in a later semester. As their pre C-scores were above average (33.2 C-points), it cannot be concluded that the overall positive effect of PBSCDs only occurred because below-average C-score students were included in the EG_1_.

A further limitation could be that the performance of the lecturer in the respective courses had an influence on the enhancement of moral competence, but this factor showed no statistically significant influence on the pre/post C-scores (*P* = 0.54). Other situational factors could also have influenced the results as fear and anxiety, or different levels of religiosity [13]. Although we tried to control the test situation and keep it constant as well as free from, e.g., exam pressure, we cannot completely exclude the influence of such factors. The phenomenon of “moral segmentation” could have occurred as well, which means that students perform differently in the two dilemma cases [2, 5]. The calculation of the C-score is not independent from moral segmentation, as the consistency across the two dilemmas plays a role in the calculation. Though we did not find relevant moral segmentation effects for our study. That means that the described changes in moral competence in the EG and the CG did not only occur due to changes in one of the dilemma cases.

Despite the limitations of the study, the insights from this pilot study for applying PBSCD as an intervention tool in ethics classes could already mean an important result considering the higher moral demands in medical practice and the concurrent regression of moral competence during medical school. We hypothesize that interactive involvement into different normative perspectives within PBSCD could encompass learner activity [31] more effectively, reduce moral distress due to inevitable tensions between conflicting norms [32] and increase the opportunities of taking responsibility [6, 13] in the process of justification. Students in the EG were confronted more strongly with a hypothetical patient and they perhaps felt more responsible because they had to consider all relevant moral aspects, to find their own specification and to weigh up on their own, instead of applying a principle of a moral theory in a formalized way as in the CG. In particular, the increased opportunity to take responsibility in PBSCDs could be an important factor for our results, because it has already been proven effective in fostering moral competence [6, 13]. The balancing of different relevant moral arguments in PBSCDs could also result in a systematical training of individuals to develop moral reasoning independent from their subjective intuitions or from a given theoretical framework. Being able to give consideration to different moral arguments and their value for a special case at the same time could also reduce moral distress and the resulting training effect could correlate with the substance of the C-score. Further research with larger and randomized samples and different instruments is needed to prove these hypotheses.

## Conclusions

Different methods of teaching ethics did not have a statistically significant influence on the moral competence of students. Yet, the effect size in this pilot study suggests that principle-based structured case discussions may improve moral competence among medical students, especially those with low and medium pre C-scores, more so than do general introductory ethics classes with theory-based case discussions. Further research with completely randomized and larger samples is necessary to prove the results of this pilot study. Future research with different instruments is also required to study the underlying effects of PBSCDs and to advance teaching methods to foster the moral competence of students with high C-scores.

## Abbreviations

aES: Absolute effect-size
CG: Control group
DIT: Defining Issues Test
EG: Experimental group
LMU: Ludwig Maximilian University
MCT: Moral Competence Test
MJI: Moral Judgement Interview
PBSCD: Principle-based structured case discussion
SD: Standard Deviation
TBCD: Theory-based case discussion
